# How musical training affects cognitive development: rhythm, reward and other modulating variables

**DOI:** 10.3389/fnins.2013.00279

**Published:** 2014-01-20

**Authors:** Ewa A. Miendlarzewska, Wiebke J. Trost

**Affiliations:** ^1^Department of Fundamental Neurosciences, (CMU), University of GenevaGeneva, Switzerland; ^2^Swiss Centre of Affective Sciences, University of GenevaGeneva, Switzerland

**Keywords:** musical training, brain plasticity, developmental neuroscience, music education, rhythmic entrainment

## Abstract

Musical training has recently gained additional interest in education as increasing neuroscientific research demonstrates its positive effects on brain development. Neuroimaging revealed plastic changes in the brains of adult musicians but it is still unclear to what extent they are the product of intensive music training rather than of other factors, such as preexisting biological markers of musicality. In this review, we synthesize a large body of studies demonstrating that benefits of musical training extend beyond the skills it directly aims to train and last well into adulthood. For example, children who undergo musical training have better verbal memory, second language pronunciation accuracy, reading ability and executive functions. Learning to play an instrument as a child may even predict academic performance and IQ in young adulthood. The degree of observed structural and functional adaptation in the brain correlates with intensity and duration of practice. Importantly, the effects on cognitive development depend on the timing of musical initiation due to sensitive periods during development, as well as on several other modulating variables. Notably, we point to motivation, reward and social context of musical education, which are important yet neglected factors affecting the long-term benefits of musical training. Further, we introduce the notion of rhythmic entrainment and suggest that it may represent a mechanism supporting learning and development of executive functions. It also hones temporal processing and orienting of attention in time that may underlie enhancements observed in reading and verbal memory. We conclude that musical training uniquely engenders near and far transfer effects, preparing a foundation for a range of skills, and thus fostering cognitive development.

## Introduction

Psychological and neuroscientific research demonstrates that musical training in children is associated with heightening of sound sensitivity as well as enhancement in verbal abilities and general reasoning skills. Studies in the domain of auditory cognitive neuroscience have begun revealing the functional and structural brain plasticity underlying these effects. However, the extent to which the intensity and duration of instrumental training or other factors such as family background, extracurricular activities, attention, motivation, or instructional methods contribute to the benefits for brain development is still not clear. Music training correlates with plastic changes in auditory, motor, and sensorimotor integration areas. However, the current state of the literature does not lend itself to the conclusion that the observed changes are caused by music training alone (Merrett et al., 2013).

In this article we briefly review the recent literature on how musical training changes brain structure and function in adult musicians and during development. We next report evidence for near and far transfer effects in various cognitive functions that are unprecedented in comparison to other long-term practice activities in childhood. Finally, we point out the important and overlooked role of other factors that could contribute to the observed cognitive enhancement as well as structural and functional brain differences between musicians and non-musicians. We propose the mechanism of rhythmic entrainment and social synchrony as factors contributing to the plasticity-promoting role of musical training that is unique to music education. The proposed mechanism of rhythmic synchronization by which musical training yields a unique advantage of transferrable skills may provide a promising avenue of research explaining the beneficial effects on a developing brain. In addition, we pinpoint the potentially important role of genetic predispositions and motivation that is rarely controlled for in the existing literature.

The review focuses on studies investigating healthy children's and adults' response to formal musical education (primarily instrumental training) in terms of neuroplasticity observed with neuroimaging techniques, as well as in behavioral effects on cognitive performance in various domains. Although we mention and acknowledge the enormous value of music therapy with the aim of restoring lost function in diseased or disabled individuals, this topic is outside the main focus of this review. Reviewing the progress in musical training research embraced in this article leads us to the promising supposition that the induced changes in brain development and plasticity are not only relevant in music-specific domains but also enhance other cognitive skills.

### Cognitive, emotional and social functions in music perception and production

Listening to music requires certain perceptual abilities, including pitch discrimination, auditory memory, and selective attention in order to perceive the temporal and harmonic structure of the music as well as its affective components, and engages a distributed network of brain structures (Peretz and Zatorre, 2005). Music performance, unlike most other motor activities, in addition requires precise timing of several hierarchically organized actions and control over pitch interval production (Zatorre et al., 2007). Music, like all sounds, unfolds over time. Thus, the auditory cognitive system must depend on working memory mechanisms that allow a stimulus to be maintained on-line to be able to relate one element in a sequence to another that occurs later. The process of music recognition requires access and selection of potential predictions in a perceptual memory system (Dalla Bella et al., 2003; Peretz and Zatorre, 2005). Unlike speech, music is not associated with a fixed semantic system, although it may convey meaning through systems such as emotional appraisal (Koelsch, 2010; Trost et al., 2012) and associative memories.

Furthermore, music is also known to have a powerful emotional impact. Neuroimaging studies have shown that musically induced emotions involve very similar brain regions that are also implicated in non-musical basic emotions, such as the reward system, insula, and orbitofrontal cortex, amygdala and hippocampus (Blood and Zatorre, 2001; Koelsch et al., 2006; Salimpoor et al., 2011; Trost et al., 2012). However, music can have a strong influence on the emotion of the listener as well as the performer: musical engagement can be experienced as highly emotional not only as in the case of stage fright (Studer et al., 2011) but also as highly rewarding (de Manzano et al., 2010; Nakahara et al., 2011). Furthermore, in a social context, making music in a group has been suggested to increase communication, coordination, cooperation and even empathy between in-group members (Koelsch, 2010). Therefore, it could easily be conceived how musical training could have a positive impact on the well-being and social development of children and adults.

Instrumental training is a multisensory motor experience, typically initiated at an early age. Playing an instrument requires a host of skills, including reading a complex symbolic system (musical notation) and translating it into sequential, bimanual motor activity dependent on multisensory feedback; developing fine motor skills coupled with metric precision; memorizing long musical passages; and improvising within given musical parameters. Music performance, unlike most other motor activities, requires precise timing of several hierarchically organized actions and control over pitch interval production (Zatorre et al., 2007). Music sight-reading calls for the simultaneous and sequential processing of a vast amount of information in a very brief time for immediate use. This task requires, at the very least, interpretation of the pitch and duration of the notes (written on the two staves of a piano score) in the context of the prespecified key signature and meter, detection of familiar patterns, anticipation of what the music should sound like, and generation of a performance plan suited for motor translation. Formal musical instruction, therefore, trains a set of attentional and executive functions, which have both domain-specific and general consequences.

### The musician's brain: plasticity and functional changes due to musical training

Given the engagement of multiple cognitive functions in musical activities, it seems natural that in highly trained musicians brain networks underlying these functions would show increased plasticity. Several recent review papers have critically assessed the effects of musical training on brain plasticity based on neuroimaging literature accumulated to this date (Herholz and Zatorre, 2012; Barrett et al., 2013; Moreno and Bidelman, 2013). Among others, it has been reported that apart from anatomical differences in auditory and motor cortices, there are structural differences (usually in the form of increased gray matter volume) also in somatosensory areas, premotor cortex, inferior temporal and frontal regions, as well as the cerebellum in the brains of musicians compared to non-musicians' (see Barrett et al., 2013). Several longitudinal studies have found a correlation between duration of musical training and the degree of structural change in white matter tracts (Bengtsson et al., 2005), including in the corpus callosum (Schlaug et al., 2005).

While it may not be surprising that structural and functional differences are found in those brain regions that are closely linked to skills learned during instrumental music training (such as independent fine motor movements in both hands and auditory discrimination), differences outside of these primary regions are particularly interesting (for instance, in the inferior frontal gyrus in Sluming et al., 2002). Such findings indicate that plasticity can occur in brain regions that either have control over primary musical functions or serve as multimodal integration regions for musical skills, possibly mediating the transfer of musical training onto other skills. For example, a recent study investigating resting-state activity measured with fMRI in musicians compared to non-musicians found that musicians have increased functional connectivity in motor and multi-sensory areas (Luo et al., 2012). This result shows that long-term musical training influences functional brain connectivity even in research designs where no task is given, and points out that for musicians' motor and multi-sensory networks may be better trained to act jointly.

**Figure 1:**
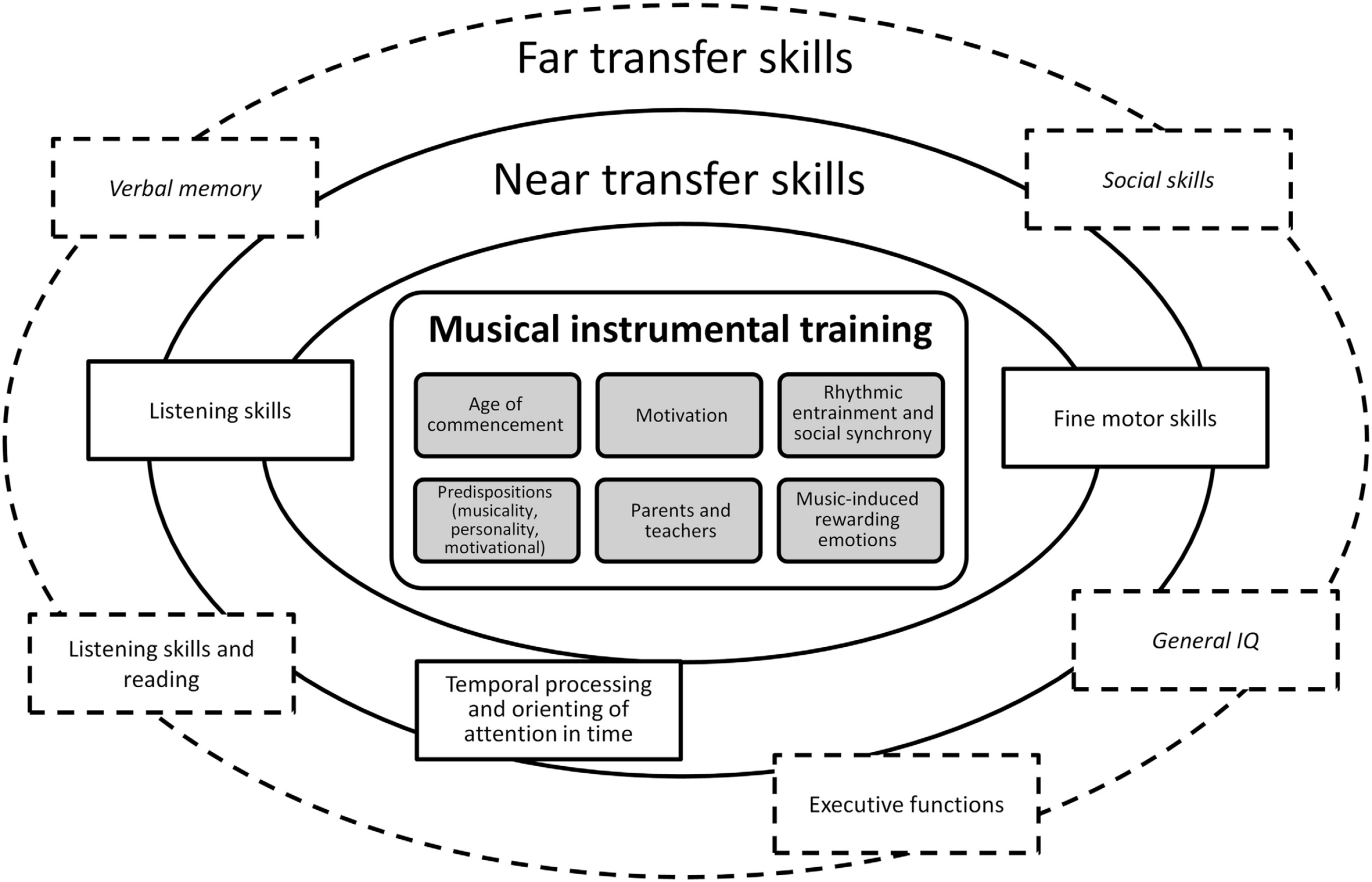
**Schematic representation of near and far transfer skills that benefit from musical instrumental training**. In the inner rectangle variables modulating the influence of musical training on cognitive development are listed (see main text, in particular section Variables Modulating Brain Plasticity via Musical Training). Near transfer skills are marked in solid rectangles and far transfer skills are marked in dashed rectangles (described in detail in section Effects on Cognitive Functions). Terms in italic indicate results inconclusive in the present state of the literature.

In the next section, we review the effects of musical training on cognitive functions and brain plasticity and discuss the role of the age at commencement. However, we note that the evidence for musical training-induced brain plasticity is largely correlational due to the number of additional variables that have not been controlled for in most of the (cross-sectional) studies (Merrett et al., 2013), and that there are unanswered questions surrounding the attribution of causal influence to musical training alone. The few random group assignment studies that have been conducted to this date, typically include a control group of participants that attend theater play, dance (Young et al., 2013), or visual arts classes (Moreno et al., 2009; Moreno and Bidelman, 2013). And while the methodological and subject-specific considerations of this matter have been discussed elsewhere (Barrett et al., 2013; Merrett et al., 2013), in section Variables Modulating Brain Plasticity via Musical Trainin we propose possible unacknowledged mechanisms that enable musicians to excel in many areas unrelated to musical skill (near- and far-transfer skills described in section Effects on Cognitive Functions). Namely, we identify the higher efficiency of attentional and memory processes engendered by rhythmic entrainment, as well as an extension of this phenomenon to social synchrony that is evoked when people sing, play music or dance together in synchrony. To summarize, in Figure 1 we propose a schema depicting the transfer skills that are enhanced by musical instrumental training, including the modulating factors discussed in sections Effects of Musical Training in Childhood and Variables Modulating Brain Plasticity via Musical Training.

## Effects of musical training in childhood

Correlational and interventional studies of children undergoing music training consistently show that they perform better in the areas closely associated with music: fine motor skill, rhythm perception and auditory discrimination. There is also strong evidence for near-transfer effects of these abilities to phoneme discrimination, as well as far-transfer effects to vocabulary, and non-verbal reasoning subsets of general intelligence tests. While near-transfer effects (transfer to tasks within the same domain) are often observed with various training programs, such as computerized executive function training (attention, working memory and task-switching) (Diamond and Lee, 2011; Jolles and Crone, 2012), far-transfer is notoriously difficult to induce and has been observed only after demanding multi-skills training such as action video games (Bavelier et al., 2010; Green and Bavelier, 2012). The reports we review in this section show that musical training also brings about promising far-transfer effects in domains such as verbal intelligence and executive functions, and may even lead to better general academic performance.

Neural development is complex and various neural processes affect plasticity. Such processes include synaptic proliferation, pruning, myelination at neurofilament and neurotransmitter levels, each of which has its own developmental trajectory (e.g., Lenroot and Giedd, 2006; Perani et al., 2010). Observing brain plasticity as years of musical training go by elucidates the way practice becomes engraved in the brain and how memory finds its reflection in brain structure. In general, studies of music learning are consistent with the animal literature indicating greater plastic changes in the brain for behaviorally relevant (e.g., associated with reward or emotional arousal) than for passive exposure to auditory stimuli (Weinberger, 2004). However, the picture is not complete until we take into account the maturational dynamics that shape the brain simultaneously with musical training. The next section introduces the concept of critical and sensitive periods in brain development which, although not exhaustively, adds to the understanding of musical training-induced neuroplasticity. The notion of “windows of opportunity” is important in that it places limits on training-related brain plasticity and hence allows to explain why certain abilities can only be developed in early childhood, which is crucial for the design of educational programs and child rearing.

### Critical and sensitive periods

It is known that plasticity is affected by how much a person actively engages in music training relatively early in their life (Knudsen, 2004). “Sensitive period” is a term applied to a limited period in development when the effects of experience on the brain are unusually strong, derived from the property of particular malleability of the neural circuits (Knudsen, 2004). During this time, the basic architecture of the neural circuits is laid out and all learning (and plasticity) that occurs after the sensitive period will cause alterations only within the connectivity patterns constraint by this framework (Knudsen, 2004). The regulation of sensitive period onset and duration is not simply by age, but by experience, and thus the presence of enriched environments may prolong sensitive periods (Hensch, 2004). For example, second language proficiency is better in individuals who have been exposed to it by the age of 11–13, marking puberty as the end of a sensitive period for language learning (Weber-Fox and Neville, 2001). In other words, the sensitive period is to some extent use-dependent (Hensch, 2004). In contrast, critical periods, are strict time windows during which experience provides information that is essential for normal development and permanently alters performance. For instance, critical period for auditory cortex plasticity ends by the age of 3–4 years in humans, as demonstrated in studies of cochlear implantation in congenitally deaf children: sensory deprivation in that time period prevents normal sensory discrimination and oral language learning (Kral and Sharma, 2012).

Not all brain regions develop with the same time course and there are unique timing and duration of critical periods across various neural systems. Sensory and motor regions enter the sensitive period earlier than temporal-parietal and frontal areas (Sowell et al., 2004), the visual cortex reaches adult levels of myelination by few months of life (Kinney et al., 1988), while in the auditory cortex myelination does not finish until 4–5 years of age (Moore and Linthicum, 2007) and white matter connectivity continues to develop until late childhood (Moore and Guan, 2001). Kral and Eggermont (2007) proposed that this extended period of developmental plasticity in the auditory cortex serves for language acquisition, wherein sensory bottom-up processing is trained by feedback from top-down cognitive processes. During this time, between ages 1 and 5, experience-dependent plasticity of the consistency of the auditory brainstem response is maximized (Skoe and Kraus, 2013).

Maturation of fiber tracts in the left frontal, temporo-occipital and anterior corpus callosum connecting the frontal lobes coincides with the development of working memory capacity, while reading ability is related to fractional anisotropy values in the left temporal lobe, as observed in children between ages of 8 and 18 (Nagy et al., 2004). Similarly, the maturation of corticospinal fibers parallels the development of fine finger movements (Paus et al., 1999). The cross-sectional area of the corpus callosum grows at least until early adulthood (Keshavan et al., 2002), while projection fibers of the posterior limb of the internal capsule (carrying sensory fibers to their processing areas in respective cortices) only approach an asymptotic point in maturation between the ages of 21 and 24 (Bava et al., 2010).

This sub-section emphasized that any intense training, including musical instrumental training in childhood, may have a different impact on brain plasticity and cognitive development depending on the age of commencement. However, many scholars of sensitive periods in brain development note that the role of motivation and attention is profound in all learning and should not be underestimated, especially during sensitive periods (Hensch, 2004). And as the example of language learning in infants shows (Kuhl et al., 2003; Kuhl, 2007), social environment and teachers may be of equally high importance.

### Effects on brain plasticity

Plastic changes in the cortical and subcortical structures of the auditory system (Gregersen et al., 2000; Wong et al., 2007; Penhune, 2011), as well as in the sensory-motor cortex (larger representation of fingers) and their functional expression depend on early age of commencement (Herholz and Zatorre, 2012), which emphasizes the role of sensitive periods in shaping training-induced plasticity (Merrett et al., 2013). Instrumental training may accelerate the gradual development of neurofilament in upper cortical layers that occurs between ages 6 and 12, underlying fast, synchronized firing of neurons (Moore and Guan, 2001; Hannon and Trainor, 2007).

Two longitudinal studies tracked the influence of musical training on behavioral and brain activity in children between the ages of five and nine. Schlaug et al. (2005) recruited 50 children who were about to begin their musical education and compared them with a group of 25 age-, socioeconomic status and verbal IQ-matched controls. At baseline, there were no pre-existing cognitive, music, motor, or structural brain differences between the instrumental and control groups as tested by functional MR scans (Norton et al., 2005). Tests performed after 14 months of musical training revealed significantly greater change scores in the instrumental group compared to the control group in fine motor skills and auditory discrimination. However, no significant changes in gray or white matter volume nor transfer effects in domains such as verbal, visual–spatial, and math were found, but the instrumental group showed a trend in the anticipated direction.

A study by Hyde et al. (2009) compared two groups of 6 years old children, one of which took private keyboard lessons for 15 months and the other spent a similar amount of time per week in a group music lesson that included singing and playing with drums and bells. Applying deformation-based morphometry to assess the differences between groups throughout the whole brain before and after the musical training revealed that children with piano lessons showed areas of greater relative voxel size in motor brain areas, such as the right precentral gyrus (motor hand area), and the midbody of the corpus callosum, as well as in the right primary auditory region, consistent with the plastic changes observed in professional musicians. Furthermore, structural brain differences in various frontal areas were observed which, however, did not correlate with improvement in behavioral performance.

This evidence demonstrates that regular musical training during the sensitive period can induce structural changes in the brain and they are unlikely only due to pre-existing morphological differences. Yet, 14 months may not be long enough to engrave statistically significant growth in white and gray matter volume (Schlaug et al., 2005), and the differences observed may potentially be confounded by parents' higher level of education (Hyde et al., 2009).

### Effects on cognitive functions

A further interesting question we explore in this section is the generalization of musical training-induced learning to other functional domains. According to the “temporal opportunity” conception of environmental stimulation during brain development, experiences in childhood and adolescence are vital to many abilities in adult life, which makes the decision of what education to provide to a child a serious matter. Is musical training a good choice? Although many longitudinal developmental studies of music education include a well-matched control group, such as another arts program, there is only limited research contrasting instrumental training in childhood with dance or sports, which could offer interesting avenues in plasticity research and aid the parents in making an informed decision. Thus, although all arts and sports programs do have beneficial effects on cognitive development (Green and Bavelier, 2008), instrumental musical training appears unique in the wide array of observed long-term effects, although there may be other factors mediating this effect (Young et al., 2013).

#### Listening skills

When comparing musically trained with untrained children, it is not surprising that differences in the performance of listening tasks and auditory processing are found. For example, it has been shown that children who benefit from musical lessons are more sensitive to the key and harmonics of Western music than untrained children (Corrigall and Trainor, 2009). More specifically, concerning pitch processing, children as young as 8, who have undergone a 6-month long music training, demonstrated increased accuracy in minor pitch differences discrimination and its electroencephalographic signature—increased amplitude of the N300 (Besson et al., 2007). No such differences were observed in the control group who has undergone an equal period of painting classes. Another recent well-controlled longitudinal study showed that children aged between 8 and 10 who benefitted from a 12-month music lesson program were better in discriminating syllabic duration and voice onset time in comparison to children who followed painting classes during the same period (Chobert et al., 2012). These results suggest thus that musical training can improve the temporal fine-tuning of auditory perception. Moreover, musicians are better at recognizing speech in noise, an ability developed through consistent practice and enhanced if music training began early in life (Parbery-Clark et al., 2009, 2011; Strait et al., 2012).

Taken together, these results suggest that musical training increases listening skills, including sound discrimination, an ability also involved in speech segmentation (Francois et al., 2013), allowing a more accurate processing of speech and voices. In line with our proposed role of rhythmic entrainment (see section Rhythm and Entrainment below), Besson et al. (2011) suggested that these differences in language processing distinguishing musicians from non-musicians may reflect a learned ability to precisely orient attention in time in order to discriminate sounds more accurately.

#### Linguistic skills

Musical sounds and all other sounds share most of the processing stages throughout the auditory system and although speech is different from music production in several dimensions (Hannon and Trainor, 2007), musical training has been shown to transfer to language related skills. For example, auditory brainstem responses to stop consonants in musically trained children as young as 3 years is more distinct, indicating enhanced neural differentiation of similar sounds that characterizes adult musicians and later translates into better ability to distinguish sounds in speech (Strait et al., 2013). While the cross-links between language and musical training have been reviewed elsewhere (e.g., Chandrasekaran and Kraus, 2010; Besson et al., 2011; Strait and Kraus, 2011, 2013), two examples include neurophysiological mechanisms underlying syntax processing in both music and language that develop earlier in children with musical training (Jentschke and Koelsch, 2009), and the transfer of musical training to pitch discrimination in speech as well as reading aloud in 8-year old children (Moreno et al., 2009).

The fact that music and language share common auditory substrates may indicate that exercising the responsible brain mechanisms with sounds from one domain could enhance the ability of these mechanisms to acquire sound categories in the other domain (Patel and Iversen, 2007; Patel, 2008). Patel argues in his OPERA hypothesis that the benefits of musicians in speech encoding are due to five mechanisms (Patel, 2011, 2013). He suggests that there is an overlap of common brain networks between speech and music, which are especially trained because music production demands high precision. Furthermore, musical activities have high emotional reinforcement potential, which stimulates these brain networks repeatedly and requires a certain attentional focus. Patel claims that these processes are responsible for the good performance of musicians in speech processing.

This benefit of musical training can not only be found in tasks of auditory perception (for example tested with the Gordon's Intermediate Measures of Music Audiation, Schlaug et al., 2005), but also in verbal abilities such as verbal fluency and memory, second language acquisition and reading abilities, demonstrating far transfer effects of musical training (for a review see Besson et al., 2011). For example, it has been shown that children with musical training performed better at the vocabulary subtest of the Wechsler Intelligence Scale for Children (WISC-III) than a matched control group (Schlaug et al., 2005; Forgeard et al., 2008). Moreover, musical training has also been associated with enhanced verbal memory (Chan et al., 1998; Ho et al., 2003; Jakobson et al., 2003).

Research in adults clearly showed that musical ability could predict linguistic skills in the second language learning. Slevc and Miyake (2006) tested 50 Japanese adult learners of English, and found a relationship between musical ability and second language skills in receptive and productive phonology, showing that musical expertise can be a benefit for learning a second language. And in young children, a study by Milovanov et al. (2008) showed that second language pronunciation accuracy correlates with musical skills.

Empirical research on children and adults suggests that musical abilities predict phonological skills in language, such as reading. For example, Butzlaff (2000) found a significant association between music training and reading skills. In another study Anvari et al. (2002) studied the relation between early reading skills and musical development in a large sample of English-speaking 4- and 5-year-olds. Learning to read English requires mapping visual symbols onto phonemic contrasts, and thus taps into linguistic sound categorization skills. In this study, both musical pitch and rhythm discrimination were tested. For the group of 5-year-olds, performance on musical pitch, but not rhythm tasks predicted reading abilities. Such a finding is consistent with the idea of shared learning processes for linguistic and musical sound categories. However, despite this negative finding in 5-year old participants, there seems to be a link between abilities of rhythm production and reading, as we elaborate in section Rhythm and Entrainment below. For example, a recent study Tierney and Kraus showed that in adolescents the ability to tap to the beat is related to better reading abilities, as well as with performance in temporal attention demanding tasks, such as backward masking (Tierney and Kraus, 2013). This difference in rhythm processing might be due to the way how rhythm perception and production was studied by Anvari and colleagues, which required short term memory abilities, whereas the task of tapping to the beat solicits rather sensorimotor synchronization, and more importantly temporal orienting of attention—an ability required also in reading.

#### Spatial and mathematical skills

A meta-analysis of 15 experimental studies by Hetland (2000) showed that music instruction enhances performance on certain spatial tasks (such as the Object Assembly subtest of the WISC) but not on Raven's Standard Progressive Matrices, which is a test of non-verbal reasoning with some visual-spatial elements. The results of correlational studies testing the association between music training and spatial outcomes show no clear-cut association, with five out of 13 studies reporting a positive correlation between music training and spatial outcomes and eight a negative, null, or mixed results. Forgeard et al. (2008), however, did not find any differences in spatial skills between children who received at least 3 years of musical training and controls. Another study (Costa-Giomi, 1999) found that children receiving piano lessons improved more than controls in visual-spatial skills but only during the first 2 years of instruction, with no differences between the groups by the end of the third year. A study with adults showed that musicians did not perform better than non-musicians in a spatial working memory task (Hansen et al., 2012). It appears, therefore, that instrumental music training may aid the acquisition of spatial abilities in children rather than bring about a permanent advantage in musicians. Finally, Schlaug et al. (2005) found no transfer effects of musical training to math skills or general intelligence in 9–11-year-olds with an average of 4 years of musical training, although the children scored higher on the vocabulary subtest of the Wechsler Intelligence Scale for Children (WISC-III), suggesting that far transfer to linguistic abilities may be the most robust one, observable already after a relatively short period of practice.

A meta-analysis of the studies investigating the influence of musical training on math performance did not show convincing evidence in favor of a transfer effect (Vaughn, 2000). Also in more recent studies no positive relation between musical training and performance in a mathematical skills tests (Forgeard et al., 2008), nor increased musicality among mathematicians has been reported (Haimson et al., 2011).

#### Executive function

The notion of executive function refers to the cognitive processes orchestrated by the prefrontal cortex that allow us to stay focused on means and goals, and to willfully (with conscious control) alter our behaviors in response to changes in the environment (Banich, 2009). They include cognitive control (attention and inhibition), working memory and cognitive flexibility (task switching).

Hannon and Trainor (2007) proposed that musical training invokes domain-specific processes that affect salience of musical input and the amount of cortical tissue devoted to its processing, as well as processes of attention and executive functioning. In fact, the attentional and memory demands, as well as the coordination and ability to switch between different tasks, which are involved in learning to play an instrument, are very large. This learning depends on the integration of top-down and bottom up processes and it may well be that it is the training of this integration that underlies the enhanced attentional and memory processes observed in the musically trained (Trainor et al., 2009). Executive functions seem thus highly solicited when learning to play an instrument (Bialystok and Depape, 2009). In fact, Moreno et al. (2011) found that even after a short-term musical training (20 days) with a computerized program children improved their executive functions tested in a go-/no-go task. Similarly, in terms of working memory capacity, a recent longitudinal study showed that children that had been included in 18-months long instrumental music program outperformed the children in the control group that followed a natural science program during the same period (Roden et al., 2013).

#### General IQ and academic achievement

Extensive amount of research on how music can increase intelligence and make the listener smarter has been carried out (Rauscher et al., 1993; Degé et al., 2011; Moreno et al., 2011). The outcome of this research shows that not music listening but active engagement with music in the form of music lessons sometimes confers a positive impact on intelligence and cognitive functions although such results are not always replicated. A major discussion in this area is whether musical training increases specific skills or leads to a global un-specific increase in cognitive abilities, measured by a general IQ score.

For children, music lessons act as additional schooling—requiring focused attention, memorization, and the progressive mastery of a technical skill. It is therefore likely that transfer skills of executive function, self-control and sustained focused attention translate into better results in other subjects, and eventually in higher scores of general IQ. General IQ is typically tested with Raven's Progressive Matrices (Raven, 1976), although various types of intelligence can also be tested on specific tests. These tests require different kinds of cognitive performance, such as providing definitions of words or visualizing three-dimensional objects from two-dimensional diagrams, and are regarded as a good indicator of mental arithmetic skills and non-verbal reasoning. For example, Forgeard et al. (2008) found that practicing a musical instrument increases the performance in the Raven's Matrices test, which could suggest that non-verbal reasoning skills are better developed in children with musical training.

Measuring intelligence implies the sensitive discussion on genetic predisposition and environmental influence, and experience-acquired abilities. Schellenberg points out that children with higher cognitive abilities are more likely to take music lessons and that this fact can bias studies in which participants are not randomly assigned to music or control conditions (Schellenberg, 2011a). Similarly, also the socioeconomic context is known to influence the probability that children get access to musical education (Southgate and Roscigno, 2009; Young et al., 2013). Controlling for this potentially confounding factor, Schellenberg (2006) reported a positive correlation between music lessons and IQ in 6–11 year olds, and showed that taking music lessons in childhood predicts both academic performance and IQ in young adulthood (holding constant family income and parents' education). In another study, two groups of 6 year-olds were tested, one of which received keyboard or singing lessons in small groups for 36 weeks (Schellenberg, 2004), and the other children received drama lessons. The latter did not show related increases in full-scale IQ and standardized educational achievement, but notably, the most pronounced results were in the group of children who received singing rather than piano lessons. Modest but consistent gains were made across all four indexes of the IQ, including verbal comprehension, perceptual organization, and freedom from distractibility and processing speed, suggesting that music training has widespread domain-general effects.

Intelligence measurements are often used to predict academic achievement. One question in this domain of research is therefore how musical activities influence academic achievement in children and adolescents. Despite initial claims that this effect may be primarily due to differences in socioeconomic status and family background, intervention studies as well as tests of general intelligence seem to show a positive association between music education and academic achievement. For example in a study by Southgate and Roscigno (2009) longitudinal data bases which include information on music participation, academic achievement and family background were analyzed. Their results show that indeed music involvement in- and outside of school can act as a mediator of academic achievement tested as math and reading skills. However, their results show also that there is a systematic relation between music participation and family background. Nonetheless, a recent study found that academic achievement can be predicted independently of socioeconomic status only when the child has access to a musical instrument (Young et al., 2013). Interestingly, this finding emphasizes that musical activities with an instrument differ from other arts activities in this respect.

Furthermore, it has been suggested that executive functions act as a mediator in the impact of music lessons on enhanced cognitive functions and intelligence. Schellenberg (2011a) had the goal to investigate in detail this hypothesis of the mediating effect of the executive functions. He designed a study with 9–12-year old musically trained and un-trained children and tested their IQ and executive functions. Schellenberg's results suggest that there is no impact of executive functions on the relation between music training and intelligence. However, other studies have reported such an influence. For example there has been evidence that musical training improves executive function through training bimanual coordination, sustained attention and working memory (Diamond and Lee, 2011; Moreno et al., 2011). Degé et al. (2011) even used a design very similar to Schellenberg's with 9–12-year old children in order to test the role of executive functions. These authors did find a positive influence of musical training on executive functions and argued that this difference of results is due to the fact that in Schellenberg's study no direct measure of selective attention was included, which supposedly plays a crucial role in music.

#### Social skills

Apart from the concept of general IQ, Schellenberg (2011b) studied the influence of musical training in children on emotional intelligence but did not find any relation between them. Moreover, another study with 7–8 year-old children found a positive correlation between musical training and emotion comprehension which disappeared, however, when the individual level of intelligence was controlled (Schellenberg and Mankarious, 2012). Also other studies with adults did not find any correlation between musical training and emotional intelligence (Trimmer and Cuddy, 2008). One study by Petrides and colleagues with musicians did find a positive correlation between length of musical training and scores of emotional intelligence (Petrides et al., 2006). There seems to be thus a still contradictory picture concerning the association between emotional intelligence and musical education. This result is interesting insofar as it could be thought that musical training could also increase social competences, given that active musical activities have shown to enhance communicative and social development in infants (Gerry et al., 2012). Moreover, a study by Kirschner and Tomasello (2009) found that in children at the age of 4 musical activities produced behaviors of spontaneous cooperation.

Another way to test social skills is to investigate the sensitivity to emotional prosody, which is a precious capacity in social communication. Studies have shown that musical training enhances the perception and recognition of emotions expressed by human voices (Strait et al., 2009; Lima and Castro, 2011), although an earlier study found that not musical training, but rather emotional intelligence predicted the recognition of emotional prosody (Trimmer and Cuddy, 2008). Thus, like with regards to emotional competence, the literature linking musical education and the recognition of emotional prosody is equivocal. The impact of musical education on social skills might therefore have to be investigated more in depth, comparing aspects such as music teaching methods in groups vs. single pupil lessons, and the role of musical activities in groups, for example in instrumental ensembles or choirs.

## Plasticity over the life-span

Musical activities can have a beneficial impact on brain plasticity and cognitive and physical abilities also later in adult life after the critical and sensitive periods in childhood (Wan and Schlaug, 2010). For example, Herdender and colleagues showed that musical ear training in students can evoke functional changes in activation of the hippocampus in response to acoustic novelty detection (Herdener et al., 2010). In general, at an advanced age, a decline of cognitive functions and brain plasticity can be observed. However, physical as well as cognitive activities can have a positive impact on the preservation of these abilities in old age (Pitkala et al., 2013). In this sense, musical training has been proposed as a viable means to mitigate age-related changes in auditory cognition (for a review see Alain et al., 2013)

It is often reported that with age fluid intelligence decreases and that this can be related to a diminishment of hippocampal volume (Reuben et al., 2011). In turn, a recent study by Oechslin et al. (2013) found that fluid intelligence is predicted by the volume of the hippocampus in musicians, which suggests that musical training could be used as a strategy to reduce age-related decline of fluid intelligence. In another study by Hanna-Pladdy and Mackay (2011), significant differences between elderly musicians and non-musicians (60–83 years) were found in non-verbal memory, verbal fluency, and executive functions. This shows as well that musical activity can prevent to some degree the decline of cognitive functions in ageing. However, these differences could be due to predisposition differences. Nonetheless, Bugos et al. (2007) performed a study in which predisposition influences were ruled out as they assigned participants randomly to two groups that received either piano lessons or no treatment. They found that persons over 60 who only began to learn to play the piano and continued during 6 months showed improved results in working memory tests as well as tests of motor skills and perceptual speed, in comparison to a control group without treatment. Dalcroze Eurhythmics, which is a pedagogic method based on learning music through movements and rhythm as basic elements has also been administered to seniors. One study showed that a treatment with this method during 6 months positively influences the equilibrium and regularity of gait in elderly (Trombetti et al., 2011). Given that falls in this population are a major risk, it is especially important to engage in training of these physical abilities at this age, which seems to be more efficient in combination with musical aspects of rhythmical movement synchronization and adaptation within a group.

Although there are promising results suggesting that older musicians compared to matched controls show benefits not only in near-transfer but also some far-transfer tasks such as visuospatial span, control over competing responses and distraction (Amer et al., 2013), the nature vs. nurture problem remains. Apart from the study of Bugos et al. (2007) who used a random-assignment design, research on the influence of musical training on plasticity and cognitive benefits in advanced ages should take into account the influence of other cognitive stimulations and overall physical fitness, which are known to play an important role in the preservation of cognitive functioning and independence in the elderly (Raz and Rodrigue, 2006; Erickson et al., 2012).

## Variables modulating brain plasticity via musical training

One challenge in assessing developmental changes in the brain due to long-term learning such as musical training is that many studies demonstrating structural brain differences are retrospective and look at mature musicians, which does not rule out the possibility that people with certain structural atypicalities are more predisposed to become musicians. If this is the case, then the distinction between innate and developed differences is rather difficult. In fact, the biggest goal for most training studies, notwithstanding musical training, is to disentangle the effects of longitudinal training and pre-existing differences or factors other than the intervention, such as gender, genetic predisposition, general IQ, socio-economic background and parents' influence. Another difficulty of interventions in young populations concerns the fact that children's brains are very inhomogeneous, and therefore comparisons, even within similar age groups, may not be very informative.

### Genetic predispositions

The musician's brain is recognized as a good model for studying neural plasticity (Munte et al., 2002). The fact that in several studies, a correlation was found between the extent of the anatomical differences and the age at which musical training started strongly argues against the possibility that these differences are preexisting and the cause, rather than the result of practicing music. On the other hand, the contamination of most longitudinal studies with children is that they are correlational, and most do not assign the subjects randomly to either musical education or a control group. As a result, the observed positive effects on cognitive functioning may not solely derive from practicing music but also from differences in motivation for learning or general intelligence, musical predispositions aside. Because general cognitive abilities (Deary et al., 2010) and personality (Veselka et al., 2009) are to some degree genetically predetermined, individual differences in these areas observed in musicians (vs. non-musicians) are unlikely to be solely a consequence of music training (Barrett et al., 2013; Corrigall et al., 2013).

The nature vs. nurture debate around musical practice-induced plasticity goes on and has begun to gain momentum as the number of neuroimaging studies continues to grow and recent genome-wide association studies have confirmed that many attributes of musicality are hereditary. Musical pitch perception (Drayna et al., 2001), absolute pitch (Theusch et al., 2009), as well as creativity in music (Ukkola et al., 2009), and perhaps even sensitivity to music (Levitin et al., 2004), have all been found to have genetic determinants. Importantly, these predispositions are typically tested for in children in a music school entrance exam. Therefore, it is fair to acknowledge that while learning a complex skill, such as playing an instrument, shapes brain function and structure, there may be additional explanatory variables that contribute to the observed differences between the brains of “musicians” and “non-musicians.”

### Motivation and the rewarding power of music

At least some components of cognitive abilities that are found to be better in the musically-trained stem from innate qualities (Irvine, 1998), but it is difficult to expect ecologically-valid intervention studies to be able to untangle this factor from the effect of training (Barrett et al., 2013). Corrigall et al. (2013) have pointed out that musically trained children and adolescents are typically good students, with high auditory and visual working memory and high IQ not necessarily due to their music education but due to genetic predispositions, which also make them more likely to take on instrumental classes. They describe how a number of individual traits, such as conscientiousness, persistence, selective attention and self-discipline that are needed in music training, could be the pre-existing qualities that facilitate learning, brain plasticity, as well as far-transfer effects.

In fact, personality trait “openness to experience,” which Corrigall et al. (2013) found to be considerably more prominent in those who took music lessons than in those who did not, is correlated with curiosity and tendency to explore, and may affect the way children learn and approach new skills such as music. This particular personality trait is genetically determined to some extent and may be also responsible for motivation to learn. Specifically, the expression of dopamine D4 receptors in the prefrontal cortex has been associated with the trait Openness/Intellect (DeYoung et al., 2011) and it is considered that prefrontal dopaminergic transmission is responsible for attentional control and working memory (Robbins, 2005). Dopamine receptors also play a major role in shaping motivation: genetic variants of the proportion of the dopamine receptors type 1 to type 2 in the striatum (Frank and Fossella, 2010), determine the tendency to learn from positive feedback as opposed to negative feedback and may thus affect intrinsic motivation—a major factor in training any complex skill in the long term.

Rewarding value of a musical activity could be one of the driving forces for brain plasticity induced by musical training. Due to dopamine's important role in long-term memory formation (e.g., Lisman and Grace, 2005; Schott et al., 2006; Rossato et al., 2009; Wimber et al., 2011), both the genetic polymorphisms suggested above and activity-induced dopaminergic transmission will have an influence on learning outcome as well as on future learning and the reinforcing quality of music learning. A positive affective experience, such as pleasure and pride derived from first music lessons will likely promote future practice and total duration of training. In practice, it is difficult to control for levels of intrinsic motivation in empirical studies of musical training, such as those conducted by Moreno and colleagues (Besson et al., 2007; Moreno et al., 2009; Moreno and Bidelman, 2013), but its role may considerably affect the long-term outcome.

Other factors that affect music performance ability are emotional support from parents and a nurturing relationship with the teacher characterized by mutual liking (Sloboda, 1993). Although these are not the focus of this article, they greatly affect a child's motivation to practice and the learning outcome, and should be taken into consideration in future studies investigating effects of musical training compared to other forms of long-term training intervention.

Variance within musicians may also be a variable contributing to the musical training effect. The level of musical training is linked to pleasurable experience when listening to music (Gold et al., 2013), due to the adopted listening style in musicians and an involvement of the musically activated reward system that is also implicated in reinforcement learning (Salimpoor et al., 2013; Zatorre and Salimpoor, 2013). However, little is known about individual variability in music-induced positive emotional responses. It is possible, for instance, that individuals who experience deeply rewarding musical emotions are drawn to taking on musical training (again, with potential genetic influences such as in individuals with William's syndrome, Levitin, 2012). Later on, pleasure from the performance of music may add to the intrinsic motivation to continue training, thus forming a self-reinforcing cycle in which a student with innate predispositions to rewarding musical emotions experiences satisfaction with his own performance which encourages the student to practice. In addition, as with any skill learning that takes years to master, a high tolerance to frustration and perseverance are personality traits that would render a student more likely to continue the training (Barrett et al., 2013).

Interestingly, musicians may differ in the level of enjoyment they derive from their artistic activity, with a particular difference between popular, jazz- and folk- vs. classical musicians. Although studies mostly concentrate on musicians trained in playing a particular instrument, the type of education they received may affect the outcome not only due to instructional differences but also through differences in motivation. One large survey conducted in the UK between 2006 and 2008 reported that folk, jazz and popular music students/artists derive more pleasure from their work than classical musicians (de Bezenac and Swindells, 2009). The non-classical musicians reported more frequent “playing for fun” and generally more enjoyment derived from group performances. One of the study's conclusions was that popular music artists tend to have higher levels of intrinsic motivation (and reportedly learning to play an instrument out of own desire) and later age at training commencement than classical musicians. The latter, who may have been confronted with higher demands for discipline and compliance in the formal educational system, tended to value technical skills higher than pleasure, and presumably had higher levels of extrinsic motivation for awards in adult career, and for teacher's praise during training. Although brain plasticity studies have so far mainly concentrated on classical music education, it may be important to note that students with classical and non-classical music education may actually differ in personality traits (such as conscientiousness, Corrigall et al., 2013), motivational goals, and these could in turn contribute to observed transfer of cognitive advantage and their functional and structural brain correlates.

The aforementioned consideration of motivation as a learning-modulating variable leads us to the question of what happens to the learning outcomes and skill transfer in children who are forced to learn to play an instrument. In this case, music training may be an unpleasant and stressful experience. Stress experienced around the learning episode may actually promote the formation of memory related to the stressor via the cortisol and noradrenergic receptor activation in the amygdala which projects to the hippocampus and prioritizes consolidation of the emotional arousal-laden stimuli (Joëls et al., 2006). However, evidence form more ecological designs shows that stress impairs word learning and recall performance in comparison to no stress (Schwabe and Wolf, 2010). This has to do with the role of the amygdala in memory formation under stress: it not only enhances the consolidation of the stress-related stimuli but also facilitates a switch toward more habitual responding (mediated by the dorsal striatum) and away from goal-directed behavior that is mediated by the medial temporal lobe and the prefrontal cortex (Schwabe et al., 2010). The equivalent of such a switch in a typical learning situation would be moving away from deep, reflective processing under supportive, non-demanding circumstances to superficial processing under test-anxiety, which profoundly affects factual memory (Fransson, 1977).

Stress derived from fear of punishment therefore affects the way we learn and often leads to worse performance than reward motivation. The effect depends on the task at hand but a negative impact has been found in the formation of spatial (Murty et al., 2011), procedural (Wächter et al., 2009) and declarative memory formation that requires cognitive processing (Schwabe et al., 2010). Although we cannot exhaustively elaborate on the literature treating motivation, learning and transfer in education research, suffice it to say that some forms of punishment motivation resulting in stress have a negative impact on learning (Lepine et al., 2004).

In the context of musical education, we suggest thus that the aforementioned influence of personality and intrinsic motivation should be taken into account in future studies. For example, in random assignment studies on the impact of musical training, participants should also be asked to declare their personal motivation to adhere to the training at least before and after the intervention. Furthermore, personality questionnaires could be incorporated to test for traits that affect the learning style (e.g., reward sensitivity, openness, perseverance). These factors could then be used as covariates in the analysis of the effect of musical training in both behavioral and neuroimaging studies. Such information would help determine the extent of the influence of personality and motivational disposition on the long-term adherence to the program as well as its outcome in terms of transfer skills. This could be particularly pertinent given the fact, that these factors could not only limit the positive effects of musical activities but even be detrimental to cognitive and emotional development if the activity represents mainly a source of stress and negative affect. In addition, this information might also help to disentangle the real impact of the training from the influence of personality and motivation.

### Rhythm and entrainment

Here we want to point at one specific aspect, which could represent an underlying mechanism of the beneficial transferrable effects of musical training. This specific feature is related to the fact that musical activities are usually based on rhythm. Most musical styles have an underlying temporal pattern that is called meter, which defines a hierarchical structure between time points (London, 2004). Ontogenetically, rhythm discrimination is observed in infants as young as 2 months of age (Trehub and Hannon, 2006). Like adults, 7-months old infants can infer an underlying beat, categorizing rhythms on the basis of meter (Hannon and Johnson, 2005), and 9-month old infants can more readily notice small timing discrepancies in strongly metrical than in non-metrical rhythms (Bergeson and Trehub, 2006).

The theory of dynamic attending suggests that rhythmical patterns in music can only be perceived because of a synchronization of attentional processes which entrain to the periodicities contained in the auditory rhythm (Jones and Boltz, 1989). In fact, neuronal populations in the visual cortex entrain to the regular rhythm of stimulus presentation which constitutes a mechanism of attentional selection (Lakatos et al., 2008). It has therefore been suggested that musical activities that imply perception and production of rhythms train attentional processes which benefits also other cognitive functions. Indeed, a recent study with children showed that musical activities increase the accuracy of produced rhythms (Slater et al., 2013), while adult musicians are significantly more accurate in reproducing rhythmic intervals (Chen et al., 2008), detecting metrical irregularities (James et al., 2012) and maintaining the rhythm when none is externally provided (Baer et al., 2013).

Entrainment is in fact a physical principle which describes the adaptation of at least two oscillating agents toward a common phase and period, which could eventually lead to perfect synchronicity between the oscillators (Rosenblum and Pikovsky, 2003). In this sense also the adjustment of behavior (own musical output, in ensemble playing, or movements, as in dance) to the perceived regular rhythm or extracted pulse can be regarded as entrainment (Fitch, 2013). Humans can also entrain multiple motor modalities, including for example body or limb motions, vocalization and even breathing and heart rate (Müller and Lindenberger, 2011; Trost and Vuilleumier, 2013). Neural populations can also be entrained by sensory stimulation (Gander et al., 2010) or motion, such as being rocked (Bayer et al., 2011).

Research on subcortical brain plasticity has used the frequency following response (FFR) as an indicator of perceptual acuity (Moreno and Bidelman, 2013). The FFR is a component of the auditory brainstem response (Tzounopoulos and Kraus, 2009) that is phase- and frequency-locked to the acoustic parameters of an auditory stimulus. In this sense the FFR represents evidence of direct neural entrainment to the sound, be it music or speech. Several studies have used this method to test training-derived plasticity in the perceptual processing of musical and vocal parameters or speech, demonstrating faster response in musical experts (Tzounopoulos and Kraus, 2009; Chandrasekaran and Kraus, 2010).

Furthermore, there is a close link between language and reading skills and the ability to perceive and produce rhythm, as widely documented by studies in children with dyslexia (Huss et al., 2011; Goswami, 2012), or with attention deficits as for example attention-deficit-hyperactivity disorder (Ben-Pazi et al., 2003), who show difficulties in rhythmic tasks. In fact, priming with a rhythmic sequence facilitates speech processing (Cason and Schon, 2012), and performance on perceptual discrimination in all sensory domains as well as motor response tasks is better when stimuli are presented isochronously (Nobre et al., 2007).

It appears thus that in musical education, daily training of the temporal processing mechanisms has a beneficial effect on other cognitive functions, such as reading, in which attention has to be guided in a specific manner. Moreover, a study by Tierney and Kraus (2013) showed that the ability to tap to a beat was associated with better performance not only in reading but also in other attention-demanding tasks which are purportedly at the basis of executive functions. Tapping to, producing or merely perceiving a rhythm in any sensory domain leads to formation of expectations that facilitates orienting of attentional resources (Bolger et al., 2013) and entrainment of various bodily and neural functions. There is also evidence that timing or temporal processing is a skill partially explaining individual variability in cognitive-speed and non-verbal ability measures—findings based on the isochronous serial interval production task (Sheppard and Vernon, 2008; Holm et al., 2011; Loras et al., 2013). And it may even support superior auditory verbal memory in musicians (Jakobson et al., 2003).

Being able to tap to an acoustic beat may be important for executive function (Tierney and Kraus, 2013) and implies coordination of movements, anticipation and sensorimotor integration. Being able to synchronize to an external rhythm while playing an instrument requires not only fine motor skills but also good auditory-motor coordination and sensorimotor integration—capacities that are also vital in planning and executing movements in general. Indeed, the functional neuroimaging signature of sensorimotor integration is increased in musicians performing a temporal synchronization task and involves increases in brain network interaction including premotor cortex, posterior parietal cortex and thalamus (Krause et al., 2010), which are also involved in attentional processes and motor planning (Coull, 2004). Furthermore, this ability of locking into temporal patterns is a skill that is useful in social communication, in which reciprocity and turn taking is essential.

The mentioned aspects of attentional guiding, forming temporal expectations, auditory-motor integration, coordination of movements and social interaction have all in common that they are based on a synchronization and adaptation of internal processes to the external rhythm of the music, or the actions of other musicians (Trost and Vuilleumier, 2013). We therefore suggest that rhythmic entrainment and ensuing honing of temporal processing play a key role in the beneficial influence of music education on the development of executive functions and far transfer effects.

Rhythmic entrainment has also been suggested as an emotion induction mechanism (Juslin et al., 2010). According to Juslin and colleagues the process where internal bodily rhythms such as respiration adapt to the external rhythm of the music contributes to the induction of an emotional reaction. Being in synchrony with the music or with the other musicians would therefore represent an emotional and often rewarding experience. As pointed out in the previous section on the influence of motivation, positive emotional experiences that activate the reward system modulate memory formation and favor brain plasticity.

Furthermore, musical activities are often social. Indeed, it has been proposed that the evolutionary function of music has always been to increase cooperation, coordination, communication, co-pathy, contact, social cognition and cohesion between the members of a group (Koelsch, 2010). It seems that one of these effects is the fact that a certain form of social synchronization is instilled, implying the respect of and adaptation to each other. In fact, in empirical studies it has often been described that acting in synchrony with a partner may increase prosocial commitment (Kokal et al., 2011), social affiliation (Hove and Risen, 2009), trust (Launay et al., 2013), cooperation (Wiltermuth and Heath, 2009) and feelings of compassion (Valdesolo et al., 2010; Valdesolo and Desteno, 2011). When playing music in a group one has to automatically synchronize to the other musicians. The state of synchrony is therefore generated naturally and is possible already in pre-school children who synchronize their drumming more easily in a social context (Kirschner and Tomasello, 2009). Learning to perform an activity in synchrony together with others is supported by the activation of the mirror neuron system (Tognoli et al., 2007; Overy and Molnar-Szakacs, 2009). We therefore suggest that this social aspect of musical training may add to the role of reward and motivation in shaping a developing brain. Moreover, learning of some skills (singing in a choir, playing in an ensemble) sets musical training apart from other social activities that do not require synchronization of actions with other group members specifically thanks to the engagement of the mirror neuron system.

Another piece of evidence for brain plasticity induced directly via rhythmic entrainment comes from the rehabilitation literature. Rhythmic auditory stimulation (RAS) is an important method in brain stimulation, which may induce short but also long term plasticity in a damaged brain (Thaut and Abiru, 2010). For example, in Parkinson patients stimulating the dopaminergic circuits in the basal ganglia leads to a reduction of movement disorder symptoms (Thaut et al., 1996; Pacchetti et al., 2000). In other neurologic diseases or acquired brain injury RAS also has beneficial effects, as the synchronization to an external beat helps to recover the coordination of movements via the stimulation of auditory-motor and sensorimotor integration (Bradt et al., 2010; Rodriguez-Fornells et al., 2012).

Neuronal populations in various areas of the brain can be entrained by external stimulation (Gomez-Ramirez et al., 2011; Nozaradan et al., 2011; Thut et al., 2011), and temporal prediction underlying the capacity for rhythmic sensorimotor synchronization (Repp, 2005; Repp and Su, 2013) has been suggested to play a general role in efficient behavior (Schwartze and Kotz, 2013). Specifically, maintenance of rhythmic regularity of neural oscillations within particular frequency bands has been suggested as a mechanism of communication across distant neural areas (Canolty and Knight, 2010; Grahn, 2012), as well as of sensory perception (Thut and Miniussi, 2009), and memory consolidation, particularly during sleep (Fell and Axmacher, 2011). Rhythmic entrainment can be conditioned using e.g., sensorimotor stimulation (Schabus et al., 2013), or induced in a sleeping brain using electric (Marshall et al., 2006) or auditory (Ngo et al., 2013) stimulation, which results in increased amount of slow waves, improved quality of sleep and better declarative memory.

Even in the animal literature interesting effect of rhythmic entrainment can be found (Rickard et al., 2005). For example, based on a series of experiments on neonate chicks, Rickard and colleagues observed that complex rhythmic auditory stimuli enhance memory by promoting moderate levels of physiological arousal through noradrenergic modulation of the memory systems (Toukhsati and Rickard, 2001; Rickard et al., 2005; Rickard, 2009). The authors concluded that it is important that the auditory stimulus contains a certain rhythmical complexity as simple metronomic beats of non-metrical rhythms do not have any memory enhancing effect (Toukhsati and Rickard, 2001). Furthermore, non-metrical (that is, consisting of tones that are not aligned with the dominant beat) music produced learning and memory deficits on a maze task in mice, while rats exposed to non-rhythmic music performed poorly in a spatial learning task (Schreckenberg and Bird, 1987; Rauscher et al., 1998). This suggests that auditory stimulations with a non-metrical rhythmical structure could compromise memory processes. Furthermore, these studies show that even in a passive listening condition performance could be improved in animals. However, we would like to emphasize that an active involvement in a rhythmic activity could amplify the effects even more, as it is known that active participation in a musical activity compared to passive listening only has a stronger effect on, e.g., executive function in the elderly (Bugos, 2010).

We propose that active engagement involving a synchronized production of motor responses is necessary for the facilitatory effect on attentional resources, movement control, auditory working memory and other functions that rely on temporal processing, as well as social synchrony. These particular aspects of musical training, absent in visual arts or theater training, contribute to the wide development of cognitive abilities and render it very different from other forms of artistic expression. We therefore suggest that a proper control group comparing music as a form of childhood intervention would be to use a group activity characterized by both rhythmic entrainment and social synchrony, such as for example team sport lessons (e.g., rowing, badminton or volleyball).

## Conclusion

In this review of the literature we show that musical training in childhood not only enhances many cognitive functions but is accompanied by neuroplastic changes in brain structure and function. Although this influence appears to be strongly potentiated when musical training takes place during sensitive periods, we have given some examples that music-induced brain plasticity does occur also later in life. In this article we wanted to point to specific factors affecting the relative value of musical education in comparison to other types of longitudinal training in childhood that require similar engagement of cognitive resources and demand a significant overall time investment. These factors include the importance of motivation, affect and social communication in music learning, as well as the potential role of rhythmic entrainment. Consequently, several issues, which have been treated in other recent review papers, remained beyond the scope of this review. Musical training results in better achievement in domains other than mere music performance, such as verbal abilities, second language learning, non-verbal reasoning and general intelligence. The advice for parents and educators is therefore clear: promote instrumental training in early childhood, as it may result in life-long advantages. However, the precise timing of the “windows of opportunity” within which particular environmental stimulation should be provided to a child to have the strongest impact, is probably before the age of seven, although the auditory system could benefit from earlier commencement, by the age of five (or even 2, Skoe and Kraus, 2013), while other structures, such as for example the white matter tracts, remain plastic well into the adulthood.

Notably, the aspect of motivation is underrepresented in the existing literature on musical training. The link between reward system activity and various forms of learning is well known: for instance, hippocampal learning (spatial, semantic, and episodic memory) is enhanced with the simultaneous activity of the reward system (involving the dopaminergic neural pathway from the ventral tegmental area and the ventral striatum Lisman and Grace, 2005), not to mention the simple reward conditioning mechanism underlying many well-documented tasks where learning and memory enhancements have been observed (e.g., Delgado and Dickerson, 2012).

Furthermore, in this review we have proposed rhythmic entrainment as a major underlying mechanism that is responsible for the beneficial effect of musical training on cognitive functions, especially with regard to the executive functions. Musical rhythms help to orient attentional processes in time, which implies a benefit for preparation and control of motor actions, and honing of temporal processing of information. We have reported several studies suggesting that these processes are also linked to cognitive functions such as reading ability and attentional focus. Furthermore, rhythmic entrainment is also regarded as an emotion induction mechanism, which again hints at the pleasurable aspect of musical activities.

On the other hand, music performance is frequently associated with a neglected and less glamorous aspect of a musician's life—performance anxiety (Kenny and Osborne, 2006). Stress also plays a role in learning, with moderate levels enhancing learning and high levels inhibiting it (Joëls et al., 2006; Howland and Wang, 2008). Unlike in a typical primary school curriculum, formal music education exposes a child to many opportunities for live solo performances, which may be stressful at times, not to mention the felt intrinsic pressure to perform well while the whole audience is listening for a possible error (Stoeber and Eismann, 2007). Naturally, exposure to such stress enables the individual to learn to overcome its disempowering impact with time, but it may be a source of childhood stress, which should be taken into consideration especially in case of highly sensitive individuals. Therefore, future studies of any long-term training intervention should ideally take into account individual differences in motivation to learn to play an instrument, as well as of subjective stress levels associated with the learning activity, as factors potentially modulating the investigated effects.

In Figure 1 we provide a summary of the near- and far-transfer skills promoted by musical training, according to the literature reviewed in section Effects on Cognitive Functions. We designate skills that are closely linked to the musical training domain, such as fine motor skills and listening as near-transfer skills. In particular, we identify temporal processing and orienting of attention in time as an ability that is perfected in musicians but that has not been explicitly described as a transfer skill. Instead, we point that this particular skill—processing of temporal information—likely underlies farther transfer skills such as reading and verbal memory. Far-transfer skills include abilities unrelated to the context of playing an instrument but that generalize to other domains, such as executive functions and linguistic skills. In the center, we listed the variables modulating the effects of musical training. These include, firstly, genetically determined predispositions (musicality, personality and motivational disposition; section Genetic Predispositions), and age at commencement (section Critical and Sensitive Periods). Secondly, we list the degree of intrinsic motivation and its affective quality (associated with punishment or reward), and the role of parents and teachers (section Motivation and the Rewarding Power of Music). And thirdly, two factors that modulate brain development through musical training itself: rhythmic entrainment and music-induced rewarding emotions (sections Rhythm and Entrainment & Motivation and the Rewarding Power of Music).

Learning to play an instrument offers a child the opportunity for creative self-expression and the development of an identity. Furthermore, musical training can be a leisure activity and a possibility to learn a form of discipline outside of the frame of the school curriculum, which gives the opportunity for rewarding experiences of self-achievement and positive reinforcement. Moreover, music education in preschool children, or first years of instrumental classes, as well as singing in a choir, has an important social component. Learning to make music together requires the respect of others and teaches implicit communicative rules and skills. In fact, it has been suggested that making music in a group might have served an evolutionary purpose of increasing communication, coordination, cooperation and even empathy within a group (Koelsch, 2010). This notion emphasizes the fact that making music in a group can be a very rewarding activity. Furthermore, social context and well-being also have a decisive influence on brain plasticity (Davidson and McEwen, 2012), which suggests that well-being induced by musical activities would in turn help shape brain functions via the mediating influence of the reward system. Therefore, we conclude that musical education starting already early in childhood offers the opportunity to tune and train the brain for important cognitive and possibly also social functions. Furthermore, it provides the child with techniques and foundations, which will probably serve as a benefit for the entire lifetime; not to mention that having learned to play an instrument in childhood may be a great source of pleasure later on in life.

## Author contributions

Ewa A. Miendlarzewska and Wiebke J. Trost have both contributed to the writing of the manuscript.

### Conflict of interest statement

The authors declare that the research was conducted in the absence of any commercial or financial relationships that could be construed as a potential conflict of interest.
